# The outbreak of diphtheria in Indonesia

**DOI:** 10.11604/pamj.2018.31.249.16629

**Published:** 2018-12-27

**Authors:** Ramadhan Tosepu, Joko Gunawan, Devi Savitri Effendy, La Ode Ali Imran Ahmad, Amrin Farzan

**Affiliations:** 1 Faculty of Public Health, University of Halu Oleo, Kendari, Indonesia; 2 Akademi Keperawatan Pemerintah Kabupaten Belitung Tanjung pandan, Sumatera Selatan, Indonesia; 3 Center of Research, Yayasan Cipta Anak Bangsa (YCAB), Sulawesi Tenggara, Indonesia

**Keywords:** Diphtheria, Indonesia, DPT-HB-Hib

## Abstract

Diptheria is commonly caused by the aerobic gram-positive bacteria, *corynebacterium diphtheria*. We herein report an unusual case of diphtheria outbreak in Indonesia in 2017 and its possible causes and current management.

## To the editors of the Pan African Medical Journal

Diphtheria is a contagious infection caused by *corynebacterium diptheria* [1] signed by sore throat, fever, and formation of the lining of the tonsils and throat [2]. In severe cases, the infection may spread to other organs such as the heart and nervous system [3]. Diphtheria still remains a public health problem, from developed countries to developing countries, as a result of globalization that enables the movement of people from one place to another [4]. This disease affects ages from 0 to 60 years [5].

To prevent the disease, Indonesia has implemented the Expanded Program on Immunization (EPI) since 1976 and has performed vaccinations with three doses of Diphtheria Pertussis Tetanus (DPT) in infants with high coverage [6]. In other words, the immunization program has been done since more than 5 decades. There are three types of vaccine for diphtheria immunization, namely vaccine Diphtheria Pertussis Tetanus-Hepatitis B-*Hemophilus Influenza type B* (DPT-HB-Hib), *Diphtheria Tetanus* (DT), and *Tetanus diphtheria* (Td), which are given at different ages. For instance, diphtheria immunization is given by primary immunization in infants (under one year) as much as three doses of DPT-HB-Hib vaccine with a distance of one month. Subsequently, advanced immunization (booster) is given to 18-month-old children with 1 dose of DPT-HB-Hib vaccine, while the first grade primary school children is given 1 dose of DT vaccine, and given 1 dose of Td vaccine for the second grade students, and then given 1 dose of Td vaccine again in the fifth grade students [7].

Unfortunately, in 2017, diphtheria outbreak occurs in Indonesia [8], and it is predicted to have a severe impact on public health, as Indonesia is the 15^th^ largest country in the world with 267 million population. In this short article, we report the cases of diphtheria disease and its possible causes and current prevention strategies in Indonesia.

**Figure 1:**
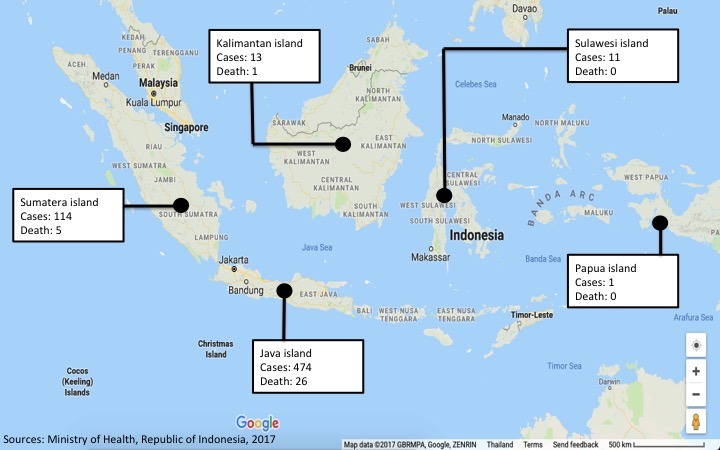
map of distribution diphtheria by Island in Indonesia

**Figure 2:**
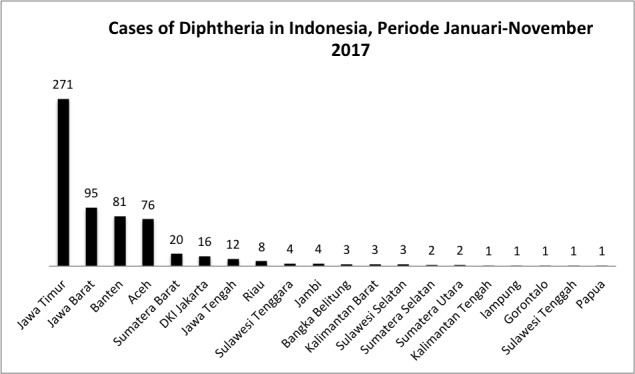
cases of diphtheria in Indonesia from January to November 2017

**Figure 3:**
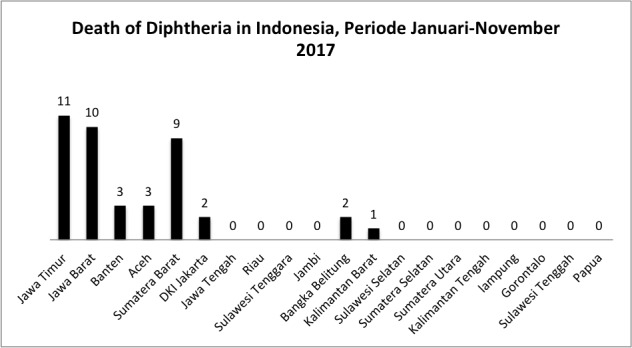
death of diphtheria in Indonesia from January to November 2017

In Indonesia, the distribution of diphtheria disease is in the five largest islands, including Java island which has 474 cases and 26 deaths, Sumatera island with 114 cases and 5 deaths, Kalimantan island with 13 cases and 1 death, Sulawesi island with 11 cases, and Papua Island with 1 case (Figure 1). And the five provinces that have the highest case of diphtheria disease from January 2017 to November 2017 were East Java (271 cases), West Java 95 cases), Banten (81 cases), Aceh (76 cases), and West Sumatera (20 cases) (Figure 2). In addition, the highest number of deaths caused by diphtheria was in East Java (11 cases), West Java (10 cases), West Sumatera (9 cases), Banten (3 cases), and Aceh (3 cases) (Figure 3).

From this report, we assume that the occurrence of diphtheria outbreaks may be associated with immunity gap, which is defined as immune deficiency among populations in a region. This deficiency is actually due to the accumulation of vulnerable groups to diphtheria, as this group had received no vaccination or incomplete immunization. However, inadequate immunization coverage causes diphtheria to increase [9]. In addition, in some areas of Indonesia, there is resistance to immunization that may lead the increase of the cases [10]. Religion is one of the objections influencing decision making on vaccination [11].

In response, the Ministry of Health of the Republic of Indonesia implements the Outbreak Response Immunization (ORI) to prevent transmission of diphtheria disease, which was implemented in children aged 1 to 19 years from December 2017 to January 2018 in schools, public health center, and other health facilities; and then the follow-up examination was performed six months later.

Additionally, regular surveillance and health workers actively identify cases quickly to prevent diphtheria [12], followed by training for health personnel on case identification, reporting and case management [4]. Until January 2018 ORI program and diphtheria prevention campaigns have been implemented, and vaccination coverage is continuously improved. [13]. However, all sectors both the government and public health professionals should work and cooperate with each other to solve the problem as well as to increase the knowledge of the community particularly the public figures and religious groups about the diphtheria disease to deal with the barriers of vaccination. It is important to emphasize that the vaccine is not for diet, but for medical and preventive purpose. According to Islamic tradition, vaccination serves to protect life, therefore *haram* ingredients could be permitted (transformation of *haram* components to halal products) to respect the principle of preventing harm (*izalat aldharar*), and public interest (*maslahat al ummah*) [14].

## Competing interests

The authors declare no competing interests.
